# Human CD206^+^ macrophages associate with diabetes and adipose tissue lymphoid clusters

**DOI:** 10.1172/jci.insight.146563

**Published:** 2022-02-08

**Authors:** Lindsey A. Muir, Kae Won Cho, Lynn M. Geletka, Nicki A. Baker, Carmen G. Flesher, Anne P. Ehlers, Niko Kaciroti, Stephen Lindsly, Scott Ronquist, Indika Rajapakse, Robert W. O’Rourke, Carey N. Lumeng

**Affiliations:** 1 Department of Pediatrics and; 2 Department of Surgery, University of Michigan Medical School, Ann Arbor, Michigan, USA.; 3 Department of Surgery, Ann Arbor Veterans Affairs Healthcare System, Ann Arbor, Michigan, USA.; 4 Center for Human Growth and Development, University of Michigan, Ann Arbor, Michigan, USA.; 5 Department of Biostatistics, University of Michigan School of Public Health, Ann Arbor, Michigan, USA.; 6 Department of Computational Medicine and Bioinformatics, University of Michigan Medical School, Ann Arbor, Michigan, USA.; 7 Department of Mathematics and; 8 Department of Biomedical Engineering, University of Michigan, Ann Arbor, Michigan, USA.; 9 Molecular and Integrative Physiology, University of Michigan Medical School, Ann Arbor, Michigan, USA.

**Keywords:** Immunology, Metabolism, Adipose tissue, Diabetes, Macrophages

## Abstract

Increased adipose tissue macrophages (ATMs) correlate with metabolic dysfunction in humans and are causal in development of insulin resistance in mice. Recent bulk and single-cell transcriptomics studies reveal a wide spectrum of gene expression signatures possible for macrophages that depends on context, but the signatures of human ATM subtypes are not well defined in obesity and diabetes. We profiled 3 prominent ATM subtypes from human adipose tissue in obesity and determined their relationship to type 2 diabetes. Visceral adipose tissue (VAT) and s.c. adipose tissue (SAT) samples were collected from diabetic and nondiabetic obese participants to evaluate cellular content and gene expression. VAT CD206^+^CD11c^−^ ATMs were increased in diabetic participants, were scavenger receptor–rich with low intracellular lipids, secreted proinflammatory cytokines, and diverged significantly from 2 CD11c^+^ ATM subtypes, which were lipid-laden, were lipid antigen presenting, and overlapped with monocyte signatures. Furthermore, diabetic VAT was enriched for CD206^+^CD11c^−^ ATM and inflammatory signatures, scavenger receptors, and MHC II antigen presentation genes. VAT immunostaining found CD206^+^CD11c^–^ ATMs concentrated in vascularized lymphoid clusters adjacent to CD206^–^CD11c^+^ ATMs, while CD206^+^CD11c^+^ were distributed between adipocytes. Our results show ATM subtype–specific profiles that uniquely contribute to the phenotypic variation in obesity.

## Graphical abstract

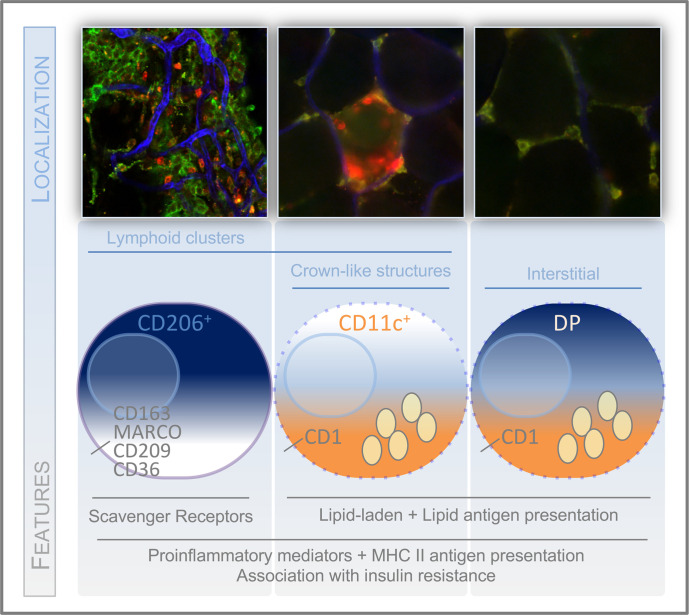

## Introduction

Obesity remains a significant public health concern, contributing to risk of cardiovascular disease, type 2 diabetes mellitus (DM), and adverse outcomes in viral infection (1). Adipose tissue dysfunction in obesity contributes to the development of metabolic disease. Stromal vascular cells (SVCs) in adipose tissue are highly responsive to nutrient excess. High-fat diet (HFD) feeding in mice induces adipocyte hypertrophy and rapidly increases the quantity of specific immune cells (2). With long-term HFD feeding, macrophages and DCs contribute to inflammation and dysregulated tissue remodeling related to metabolic dysfunction (3–5).

Skewed proportions of adipose tissue macrophage (ATM) subtypes, such as increased proinflammatory ITAX^+^ (CD11c^+^) ATMs, contribute to insulin resistance in mice (4), but the role of human ATMs and subtypes in obesity remains unclear. Human ATMs increase in obesity, and a proinflammatory type is reported to correlate with metabolic dysfunction, though various M1-like, M2-like, and mixed M1/M2 phenotypes have been observed (6–10), complicating stratification and interpretation of their function in human adipose tissue.

One challenge common to the study of ATMs and their subtypes in obesity is unambiguous pan-cell type identification. ATM surface markers overlap significantly with other myeloid cells, including monocytes and DCs (11). Discovery of unique markers will be important for defining ATMs overall and determining subtype activation states in obesity (12, 13). Accumulating data in myeloid cell populations have identified new markers for pan-stratification, and these data and prior work in mice and humans have identified FCGR1 (CD64) as a useful marker specific to ATMs (5, 11, 14). Recent single-cell transcriptomics studies on mouse and human SVCs in obesity (12, 13) enhance these data and show promise for marker discovery through unsupervised clustering. However, interpretation of functionally relevant populations from these data remains a significant challenge. Thus, data on human ATM subtypes in DM remains incomplete.

Here, we sought to improve understanding of human ATM profiles in obesity and how they relate to DM. We examined CD64^+^ ATMs from visceral adipose tissue (VAT) and s.c. adipose tissue (SAT) from a bariatric surgery cohort with obesity, evaluating correlations with clinical metrics, divergence in CD64^+^ human ATM subtypes with stratification based on classical markers CD11c and MRC1 (CD206), and alignment of ATM subtype gene expression with existing data.

## Results

### Visceral CD206^+^ ATMs correlate with metabolic dysfunction.

**Figure 1:**
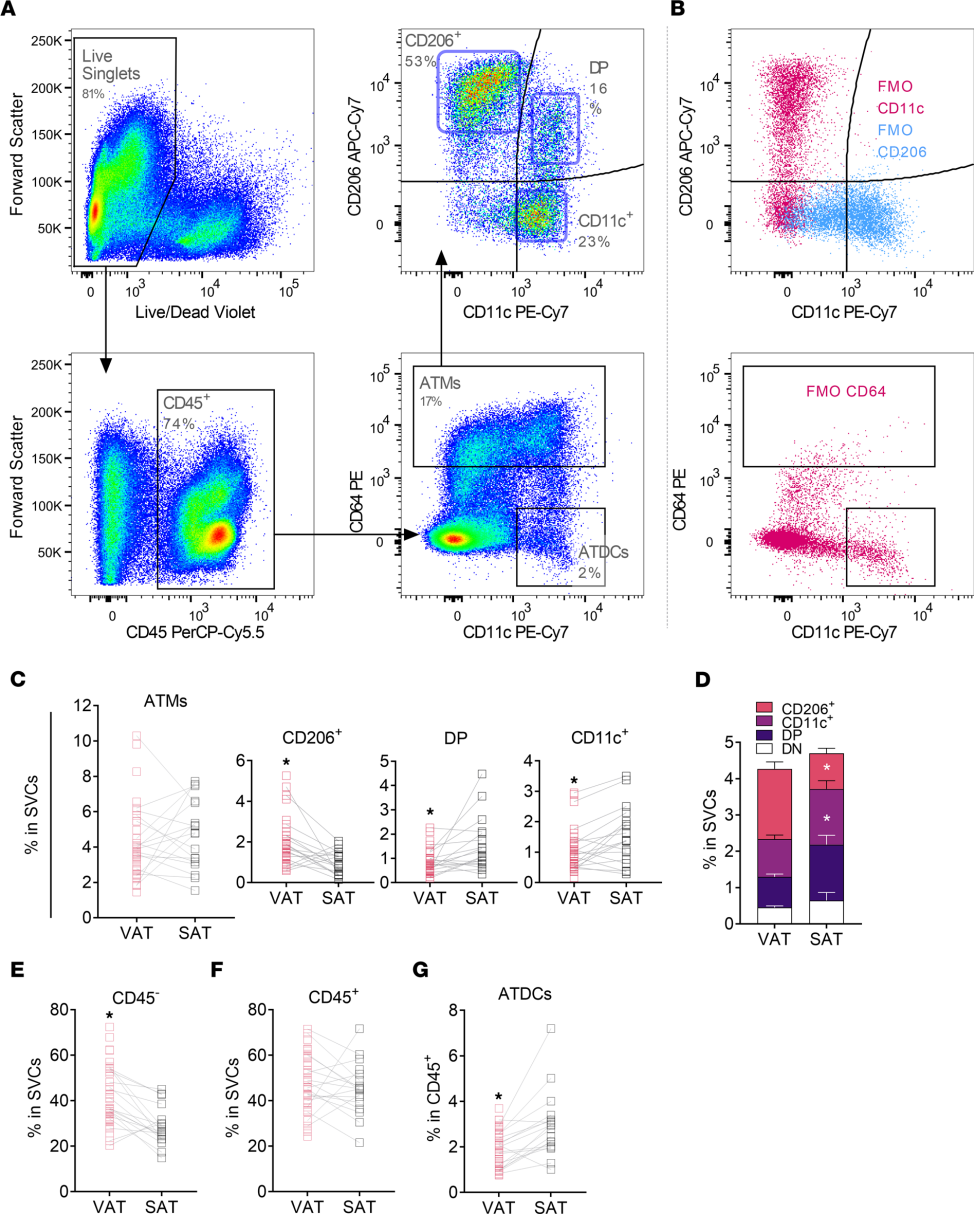
Human ATMs in VAT and SAT. (**A**) Gating scheme used to identify and isolate adipose tissue macrophages (ATM) and adipose tissue DCs (ATDC). (**B**) Three fluorescence minus one (FMO) controls which excluded immunostaining for CD11c (FMO CD11c), CD206 (FMO CD206), or CD64 (FMO CD64). (**C**) Variation in ATM frequencies between VAT and SAT depots. (**D**) Stacked bar graph showing proportions of ATM subtypes in VAT and SAT. (**E** and **F**) Frequency of CD45^–^ or CD45^+^ cells within SVCs. (**G**) Frequency of ATDCs within CD45^+^ cells. Nonparametric paired *t* test (Wilcoxon matched-pairs signed-rank test); *P < 0.05; VAT, *n* = 36; SAT, *n* = 18. DP, double positive; DN, double negative.

In prior work, we stratified human ATM subtypes and adipose tissue DCs (ATDCs) in VAT and SAT by flow cytometry, using the markers CD64, CD206, and CD11c (5). CD64 is macrophage specific and provides a clearer separation of ATMs and ATDCs, which may have overlapping presence of ITAM (CD11b), CD11c, CD68, and AGRE1 (F4/80) (5, 11). Here, we used CD64 to identify human ATMs more clearly and determine whether they correlated with DM and metabolic dysfunction in obesity in bariatric surgery patients. Within PTPRC^+^ (CD45^+^) SVCs, we identified CD64^+^ ATMs and ATM subtypes CD206^+^CD11c^−^ (CD206^+^), CD206^+^CD11c^+^ (double-positive [DP]), and CD206^−^CD11c^+^ (CD11c^+^), as well as CD64^−^CD11c^+^ ATDCs (Figure 1, A and B); this was accomplished using a stratification approach justified by prior literature and findings in mice (5, 9, 11).

Total ATMs had similar frequency in VAT and SAT of obese participants (Table 1), but differences were observed for ATM subtypes (Figure 1, C and D; endothelial cells were greater in VAT). CD206^+^ ATMs were 1.9-fold higher in VAT, with a mean of 1.9% of SVCs, while DP and CD11c^+^ ATMs were 1.8- and 1.4-fold higher, respectively, in SAT, each with a mean of 1.5% of SVCs. CD45^–^ SVCs, including preadipocytes and endothelial cells, were greater in VAT (Figure 1E). For CD45^+^ immune cells, similar frequencies were observed across VAT and SAT, while ATDCs were lower in VAT (Figure 1, F and G). Next, we investigated whether SVC frequencies correlated with age, BMI, markers of metabolic dysfunction, and blood lipoproteins in VAT and SAT. In VAT, ATMs and the CD206^+^ and DP ATM subsets correlated positively with HbA1c, fasting blood glucose and criteria for metabolic syndrome (Table 1 and Supplemental Figure 1A; supplemental material available online with this article; https://doi.org/10.1172/jci.insight.146563DS1). ATM frequency did not correlate with clinical measures in SAT, though SAT ATDCs correlated negatively with HbA1c and fasting blood glucose (Supplemental Figure 1B). By multiple regression, VAT total ATMs, CD206^+^ ATMs, and DP ATMs were significant predictors of HbA1c, adjusting for age, sex, and BMI (Supplemental Figure 1C).

### VAT CD206^+^ ATMs are increased in obese individuals with DM compared with those of normal weight and NDM individuals.

**Figure 2:**
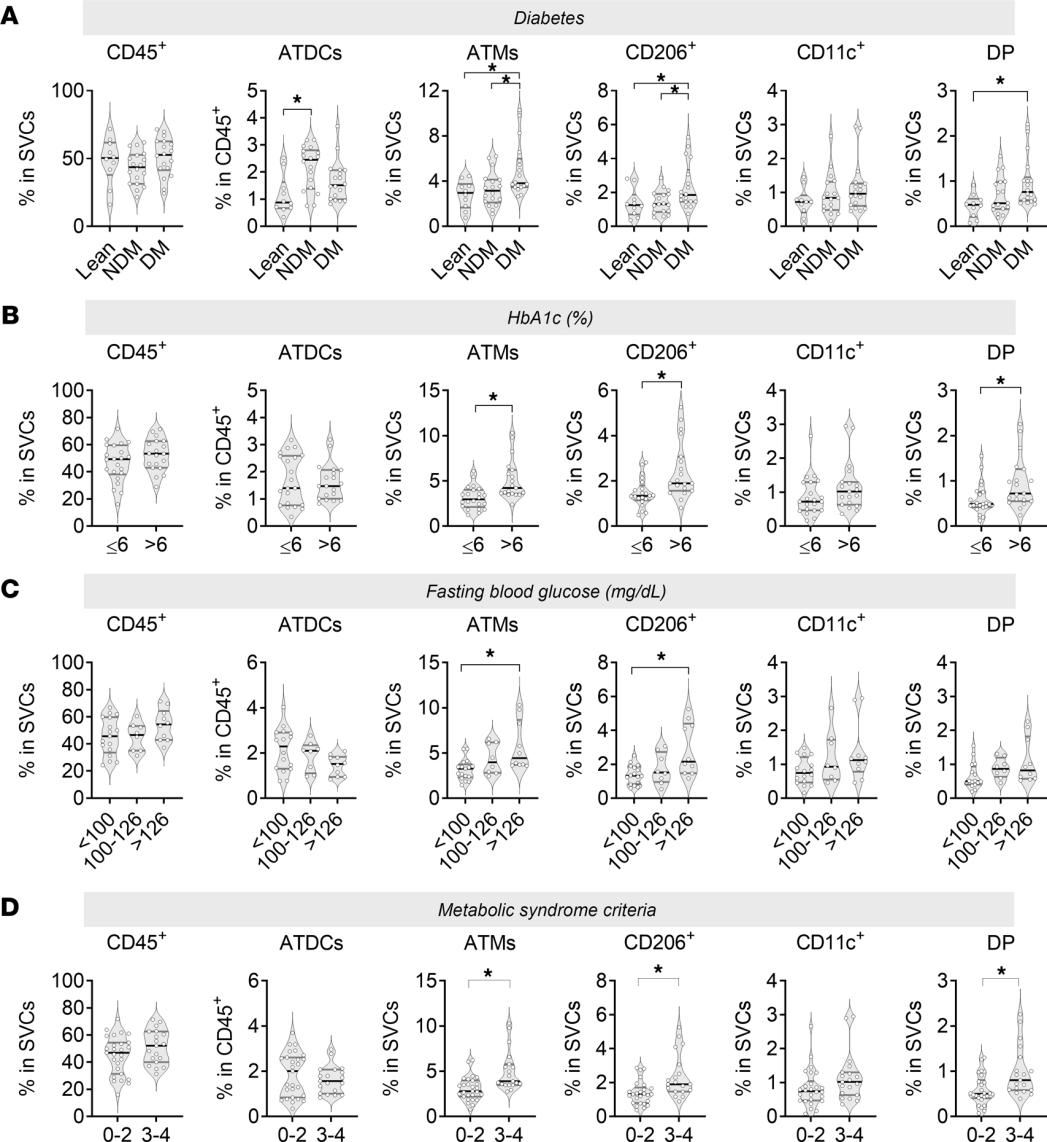
VAT SVC frequencies of patients stratified by diagnoses or clinical criteria. (**A**) Patients stratified into lean, obese NDM, or obese DM. (**B**) Patients stratified into HbA1c (%) less than or greater than 6%. (**C**) Patients stratified by fasting blood glucose (mg/dL) into groups at low risk (<100 mg/dL), considered at risk (100–126 mg/dL), and considered diabetic (>126 mg/dL). (**D**) Patients stratified by a lower (0–2 criteria) or a higher (3–4 criteria) number of criteria for metabolic syndrome (MetS). Lean: *n* = 18; obese: *n* = 10. *P < 0.05, using unpaired *t* test with Welch’s correction for 2 groups or 1-way ANOVA with Holm-Sidak adjustment for multiple comparisons. Boxes span the 25th–75th percentiles with the mean, and whiskers are minimum to maximum.

To determine whether SVC frequencies grouped with categorical clinical criteria, we stratified participants based on diagnosis of DM, metabolic measures, and number of metabolic syndrome criteria. ATMs and ATDCs from obese non-DM (NDM) and obese DM patients were compared with each other and with cells from lean patients (BMI < 30). VAT ATDCs were higher in frequency in obese NDM samples compared with lean samples and trended higher than obese DM samples (Figure 2A). We observed significantly increased total VAT ATMs in obese DM participants compared with lean and obese NDM participants, and it was related to elevated quantity of CD206^+^ and DP ATM subtypes (Figure 2A). No differences between groups were observed for CD11c^+^ ATMs. Calculating the ratio of SVC components between VAT and SAT per individual demonstrated that enrichment in VAT of total and CD206^+^ ATMs was seen in DM individuals (Supplemental Figure 1D). Elevated total ATMs, CD206^+^ ATMs, and DP ATMs were likewise observed for obese participants with HbA1c greater than 6%, fasting blood glucose above 126 mg/dL, and 3–4 criteria for metabolic syndrome (Figure 2, B–D). Examining SAT, we observed that ATDCs were lower in participants with DM, HbA1c greater than 6%, fasting blood glucose above 126 mg/dL, and 3–4 criteria for metabolic syndrome (Supplemental Figure 1E). We further stratified NDM and DM participants by sex and observed that the increase in frequency of CD206^+^ ATMs was strongest in male participants (Supplemental Figure 1F), supporting hypotheses that sex-specific differences contribute to immune cell responses in obesity (15, 16). In summary, our data show elevated VAT CD206^+^ ATMs and DP ATMs, and slightly reduced ATDCs in SAT in individuals with obesity and DM compared with obese NDM and normal weight individuals.

### Distinct transcriptional signatures in CD206^+^ ATMs.

**Figure 3:**
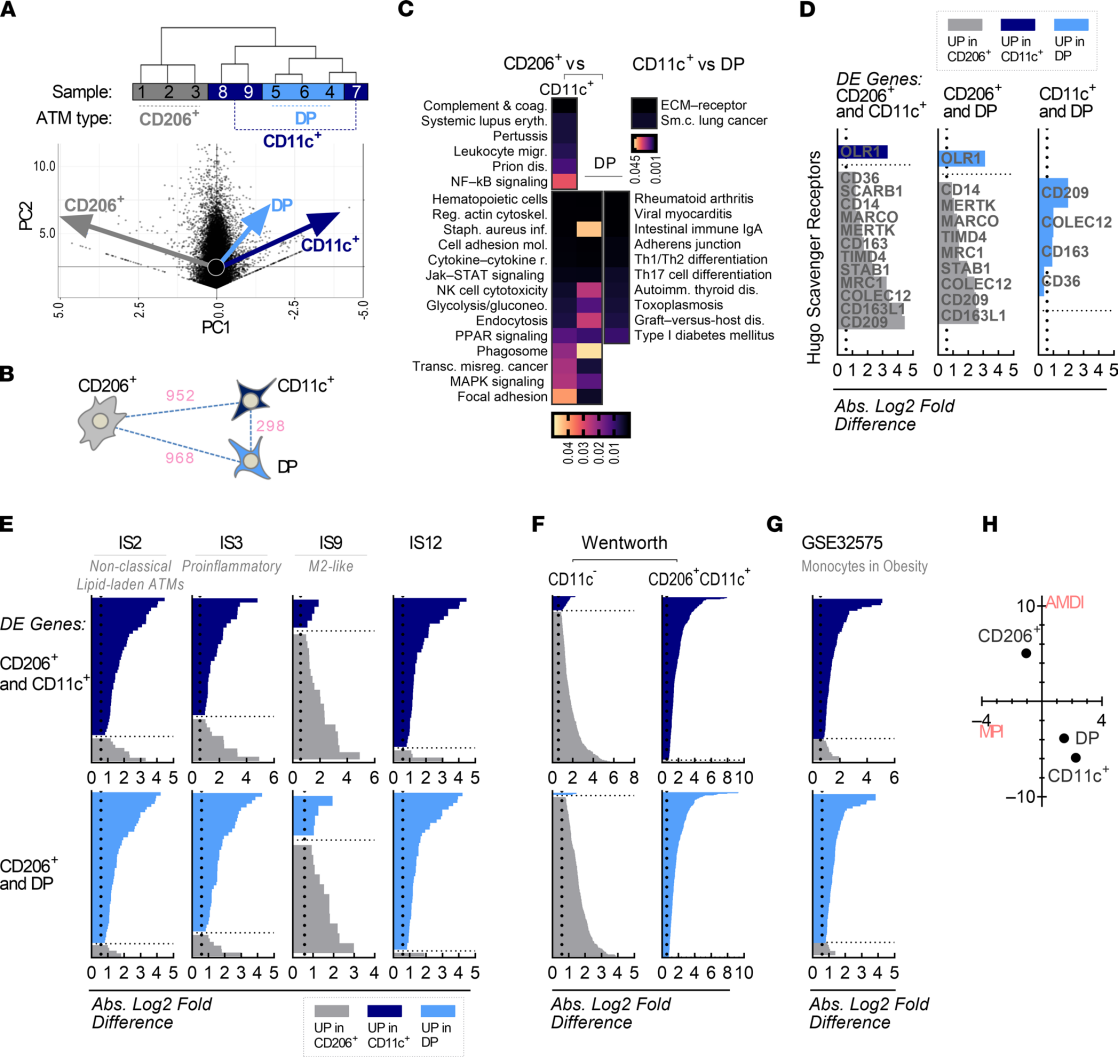
CD206^+^ ATMs are distinct from other subtypes. (**A**) Dendrogram based on the top 500 variably expressed genes (left) and PCA (right) of human ATM subtypes. The 3 ATM subtypes were each isolated from 3 patient samples with obesity (2 DM, 1 NDM) for a total of 9 analyzed cell populations. (**B**) Distance diagram between ATM subtypes based on DE genes for each comparison. (**C**) Top pathways distinguishing ATM subtypes. (**D**) Scavenger receptor–related gene expression in ATM subtypes. (**E**) Overlap of ATM subtypes with Vijay et al. data (13). Fold difference in expression for each comparison is shown, with gray bars showing expression in CD206^+^ ATMs, dark blue in CD11c^+^ ATMs, and light blue in DP ATMs. Overlap was defined as a higher percentage of mutual genes than the overall mean percentage of mutual genes across all 5 Vijay populations. (**F**) Overlap of ATM subtypes with Wentworth et al. data (9). (**G**) Overlap of ATM subtypes with monocytes in obesity (GSE32575). (**H**) MacSpectrum analysis of ATM subtypes. MPI, macrophage polarization index; AMDI, activation induced differentiation index.

To better understand ATM gene expression signatures in obese adipose tissues, we flow sorted the 3 ATM subtypes and performed RNA-Seq on each population. By principal component analysis (PCA) and dendrogram analysis of overall signatures, CD206^+^ ATMs were the most unique and distant from CD11c^+^ and DP ATMs, which were intermixed and adjacent to each other (Figure 3, A and B). Consistent with this result, a higher number of differentially expressed (DE) genes was found for CD206^+^ versus CD11c^+^ and for CD206^+^ versus DP ATMs than for CD11c^+^ versus DP ATMs (Supplemental Figure 2A). Pathway analysis also showed a greater number of significantly different pathways comparing CD206^+^ with CD11c^+^ (35 pathways, FDR ≤ 0.05) and DP ATMs (42 pathways), while only 2 pathways differed between CD11c^+^ and DP ATMs (Figure 3C). DE genes and transcription factors with top fold differences among subtypes are shown in Supplemental Figure 2, B–F.

Significant differences in scavenger receptor (SR) expression in subtypes (Figure 3D and Supplemental Figure 3A) hint at functional differences in phagocytic activity, as these characteristics have been implicated in obesity-related adipose tissue dysfunction (17–20). Most of examined SRs were expressed more highly in CD206^+^ ATMs than in DP or CD11c^+^ ATMs, suggesting a unique profile for internalization pathways that is not identified by the CD206 marker alone. We also identified a subset of SR genes, *CLEC7A*, *LY75*, and *OLR1*, that were expressed more highly in DP and CD11c^+^ ATMs.

### Overlap and clues on ontogeny of human ATM subtypes.

To better understand ATM transcriptomic data in the context of existing data, we evaluated the overlap between DE genes increased in each ATM subtype and 5 putative ATM clusters from a recent single-cell RNA-Seq study by Vijay and colleagues (13). In agreement with our prior observations, CD206^+^ ATMs aligned most strongly with IS7 and IS9 subsets, which Vijay et al. identify as having M2-like gene expression, while CD11c^+^ and DP ATMs aligned most strongly with IS2, IS3, and IS12 subsets (Figure 3E).

We next examined overlap of our ATM subtypes with subtype microarray gene expression data by Wentworth and colleagues (9). Wentworth et al. used a similar sorting approach but identified ATMs based on CD14 and stratified into 2 subtypes: CD11c^–^ (CD206^+/–^) and CD11c^+^CD206^+^, finding the latter associated with insulin resistance. Data were aligned with Wentworth signatures using DE genes among ATM subtypes with greater than a 5-fold difference and expression greater than 10 fragments per kilobase of transcript per million mapped reads (FPKM). CD206^+^ ATMs aligned with Wentworth CD11c^–^ ATMs more closely than the other subtypes, while CD11c^+^ and DP ATMs aligned more closely with Wentworth CD11c^+^CD206^+^ ATMs (Figure 3F). These data support the unique identity of the CD206^+^ ATMs in our study compared with the other 2 subtypes, and they support CD206^+^CD11c^–^ and CD206^+^CD11c^+^ ATMs having distinct functional phenotypes.

In mice, yolk sac–derived resident ATMs have been identified that are locally maintained at steady state and align with an M2-like phenotype versus CD11c^+^ ATMs that are recruited from circulating monocytes (2, 21, 22). The ontogeny of human ATMs is not well understood, but we hypothesized that the CD206^+^ ATMs are likewise a resident, SR-rich M2-type, while CD11c^+^ and DP ATMs originate from recruited monocytes. If CD11c^+^ and DP ATMs are monocyte derived, we reasoned that their gene expression would align more closely than CD206^+^ ATMs with monocyte gene expression. We therefore examined overlap with human monocytes in obesity (GSE32575; ref. 23). Indeed, monocyte genes that were also DE among subtypes were overrepresented in CD11c^+^ and DP ATMs, in support of a lineage relationship (Figure 3G and Supplemental Figure 2G). To further explore these profiles, we used MacSpectrum to evaluate ATM gene expression in terms of polarization (macrophage polarization index [MPI]) and differentiation (activation induced differentiation index [AMDI]) (24). We included all genes that were DE for at least 1 comparison among the 3 subtypes, excluding genes with FPKM < 10. Plotting the outputs for each subtype as MPI (*x* axis) by AMDI (*y* axis) (Figure 3H), CD206^+^ ATM indices were (–1.0, 5.0) in the upper left quadrant, suggesting a lower proinflammatory and more differentiated type. Consistent with earlier analyses, CD11c^+^ and DP ATMs were close to each other in the lower right quadrant with indices (2.3, –5.9) and (1.5, –3.9), respectively, consistent with a less differentiated, more proinflammatory type.

### Increased antigen presentation genes in DM with mixed expression in ATMs.

**Figure 4:**
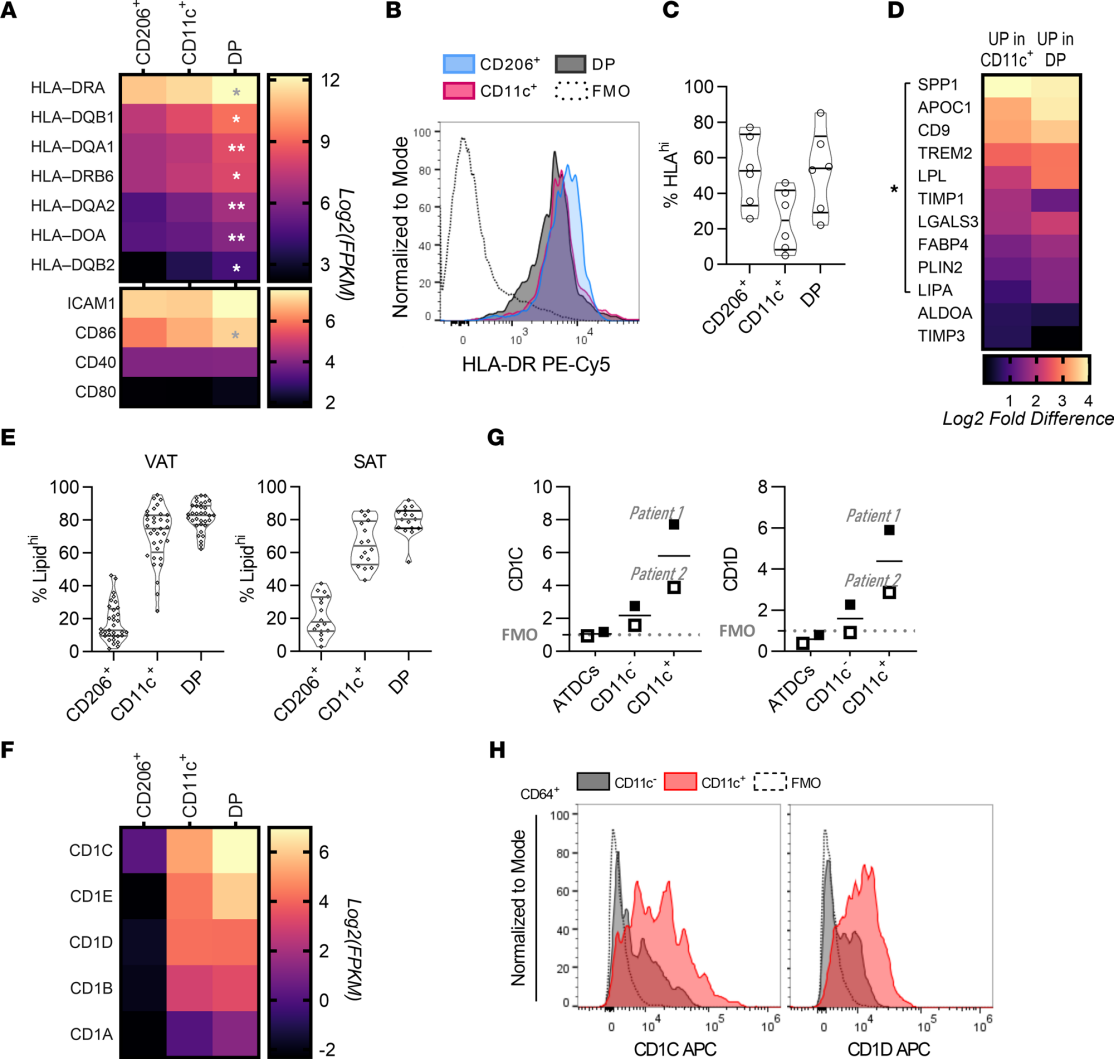
CD206^+^ ATMs have distinct HLA expression and lipid accumulation. (**A**) Heatmap of HLA-D and costimulatory gene expression in ATM subtypes (log_2_ transformed FPKM). (**B**) Surface HLA-DR in ATM subtypes by flow cytometry (counts normalized to mode). (**C**) Frequency of ATM subtypes with high surface expression of HLA-DR. (**D**) Expression of literature-curated lipid-laden macrophage markers in CD11c^+^ and DP ATMs compared with CD206^+^ ATMs. * indicates DE in the given ATMs compared with CD206^+^ ATMs. (**E**) Frequency of lipid-laden cells within ATM subtypes. (**F**) Heatmap of expression of CD1 lipid antigen presentation genes in ATM subtypes. (**G** and **H**) Flow cytometry analysis of CD1C and CD1D on ATM subtypes and ATDCs.

Insulin resistance has been associated with ATM MHC II antigen presentation in murine obesity (25, 26) and in human CD11c^+^ ATMs (9), so we examined antigen presentation–related genes *HLA-D*, *CD40*, *CD80*, *CD86*, and *ICAM1* in ATM subtypes. All ATMs expressed HLA-D genes (Figure 4A and Supplemental Figure 3B), with the highest expression in DP ATMs. To evaluate protein-level data, we performed flow cytometry for HLA-DR surface expression in SVCs from 6 patients. All 3 subtypes expressed HLA-DR in the mid to high range across patients, though HLA-DR^hi^ cells were more highly represented in CD206^+^ and DP ATMs (Figure 4, B and C). Together, these data suggest that all subtypes may participate in antigen presentation, with DP ATMs showing a consistently high number and surface expression of HLA-D genes.

### Lipid-laden ATMs and markers of lipid antigen presentation.

Different markers and anatomical locations have been attributed to human lipid-laden ATMs versus non–lipid-laden ATMs (2, 12, 27, 28). We determined overlap of ATM subtypes with markers found in the literature to be associated with lipid-laden ATMs (e.g., *TREM2*, *CD9*, *LPL*, *FABP4*) and found that CD11c^+^ and DP ATMs expressed all markers more highly than CD206^+^ ATMs (Figure 4D and Supplemental Figure 3C). Consistent with this result, by flow cytometry, CD11c^+^ and DP ATMs were high in neutral lipid content while CD206^+^ ATMs were low in neutral lipids (Figure 4E). Given their high lipid content, we hypothesized that, in DP and CD11c^+^ ATMs, markers of lipid antigen presentation may also differ, and indeed *CD1A*-*E* were significantly increased compared with CD206^+^ ATMs (Figure 4F and Supplemental Figure 3D). Flow cytometry results confirmed higher expression of CD1C and CD1D at the protein level in CD11c^+^ ATMs compared with CD11c^–^ ATMs (Figure 4, G and H, and Table 2).

### Macrophage signatures in DM VAT and SAT.

**Figure 5:**
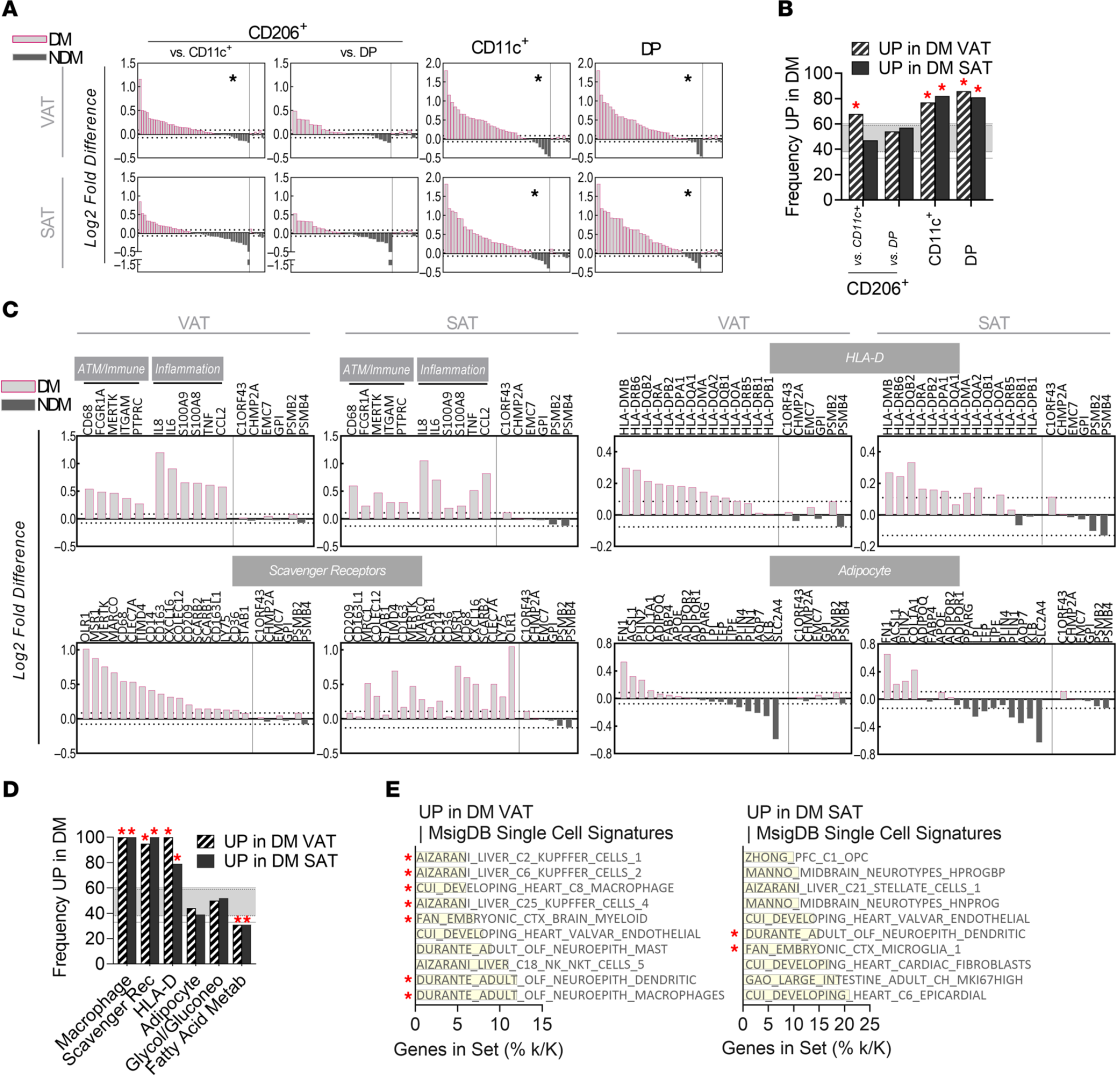
Gene expression signatures in DM and NDM adipose tissues. (**A**) Gene expression signatures unique to human ATM subtypes, queried in DM and NDM adipose tissues. Within each plot, to the right of the dividing vertical bar are housekeeping genes used to set a standard reference point for low variance in adipose tissues. Dotted lines show the largest positive and negative fold value for the housekeeping genes. (**B**) Quantification of frequency of genes within the gene sets that were increased in DM adipose. Random genes were determined through 10 iterations of random ranking and selection of the top 20 genes, followed by calculation of the frequency of genes increased in DM adipose. (**C**) Expression of gene sets representing macrophages and inflammation, scavenger receptors, and HLA-D in DM and NDM adipose tissues. An adipocyte signature exemplifies expression that was not biased toward DM. (**D**) Quantification of frequency of genes within the gene sets that were increased in DM adipose. Random genes were determined through 10 iterations of random ranking and selection of the top 20 genes, followed by calculation of the frequency of genes increased in DM adipose. Shaded band indicates the SD of the random samples of genes increased in DM VAT (band with solid lines) and in DM SAT (band with dotted lines). (**E**) Top 10 single cell signatures based on query of DE genes from DM VAT (top) and DM SAT (bottom), ranked by *q* value. Asterisks highlight signatures related to myeloid cells. Unless otherwise defined, asterisks indicate deviation of a gene set from the range of the random gene sets

We queried bulk RNA-Seq data from human DM and NDM adipose tissues (29) for gene sets that were unique to each ATM subtype, reasoning that a bias toward greater frequency of genes increased in DM represents an enrichment in that subtype’s signature. To establish a threshold for fold change in low-variance genes in adipose tissue, we included a set of 6 housekeeping genes, with dotted lines showing their maximum positive and negative fold changes (Figure 5, A and C) (30). The signature of CD206^+^ ATMs was enriched in DM VAT, while CD11c^+^ and DP ATMs were enriched in DM VAT and DM SAT (Figure 5, A and B). To additionally control for chance variation in gene sets toward DM enrichment, we selected 10 random sets of 20 genes and calculated the frequency of genes that were increased in DM adipose tissue. Figure 5, B and D, shows the range of this random variation along with the calculated frequency of genes in each gene set that were increased in DM samples.

We next queried DM and NDM tissues for enrichment of macrophage, immune cell, and proinflammatory markers. Our analyses suggest significantly increased expression of these signatures in DM tissue (Figure 5, C and D). SRs and HLA-D genes were also higher as a whole in DM VAT and SAT than NDM, while adipocyte genes remained unbiased (Figure 5, C and D). As an additional comparison between DM and NDM tissues, 2 other metabolism-related gene sets were evaluated, glycolysis/gluconeogenesis and fatty acid metabolism. While the glycolysis/gluconeogenesis gene set was within the range of random variation, the fatty acid metabolism gene set was overall reduced in both VAT and SAT DM samples (Figure 5D). Finally, we queried DE genes that were increased in DM samples against single-cell signatures. Among the top 10 results screened by *q* value, for DM VAT, 7 of 10 signatures were clearly macrophage or myeloid cell related, whereas for DM SAT, only 2 of 10 signatures were myeloid cell related, consistent with an elevated quantity of myeloid cells in human DM VAT (Figure 5E).

### Proinflammatory cytokine production in CD206^+^ ATMs.

**Figure 6:**
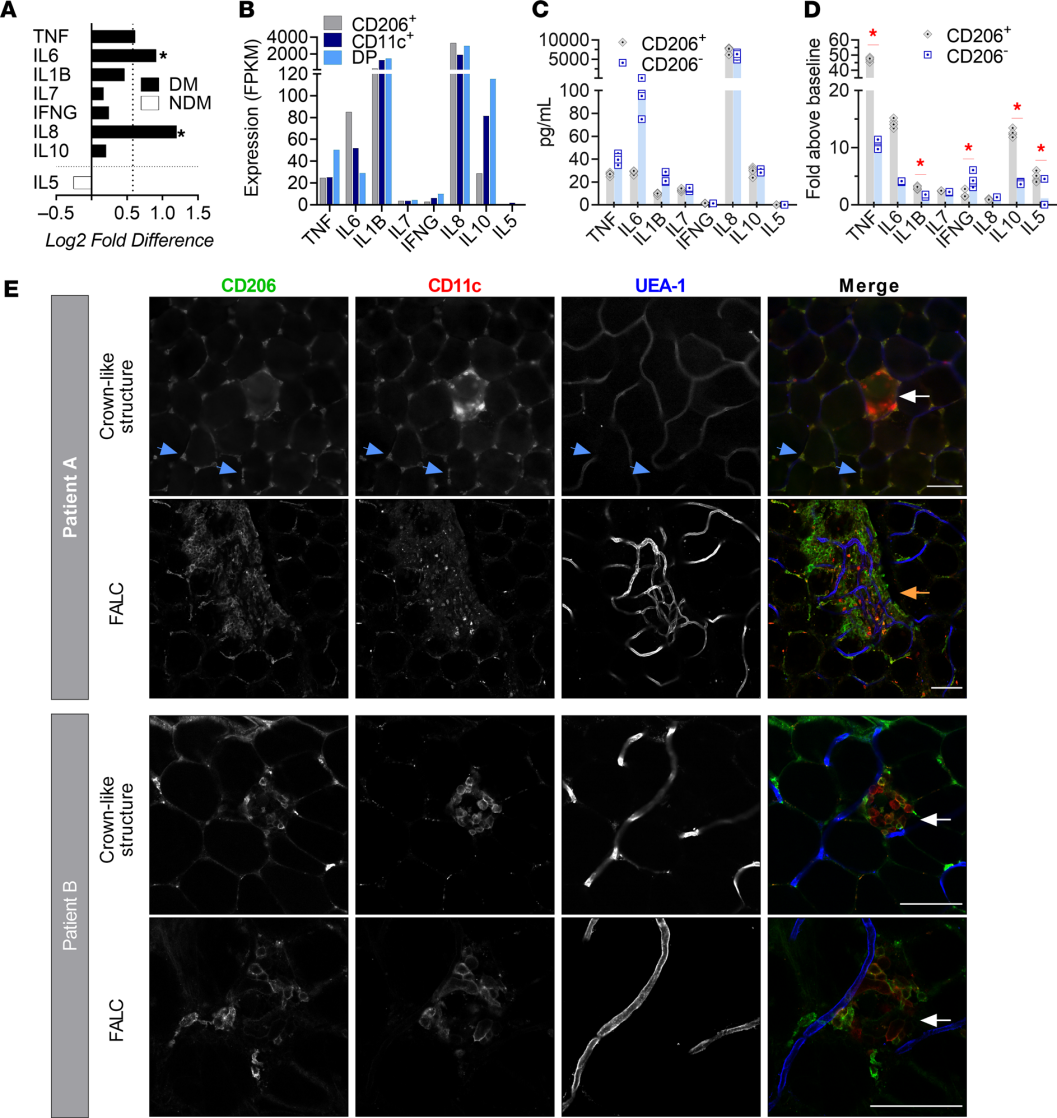
ATM subtype cytokine secretion and localization. (**A**) Expression of cytokines in NDM and DM obese whole visceral adipose tissue. Data are shown as mean FPKM log_2_-fold difference. (**B**) Expression of cytokines in CD206^+^ and CD11c^+^ ATMs by RNA-Seq. (**C**) Baseline cytokine secretion from sorted CD206^+^ or CD206^−^ VAT ATMs, shown as mean concentration. (**D**) Cytokine secretion from sorted, LPS-stimulated CD206^+^ or CD206^−^ VAT ATMs. Each replicate was normalized to baseline, and measures are shown as mean fold over baseline ± SD. (**C** and **D**) Replicates: *n* = 3–4 wells per measure. Raw data were analyzed by 2-way ANOVA with Sidak’s multiple comparison test. (**E**) Immunostaining for CD206 (green) and CD11c (red) in 2 human visceral adipose tissue samples in obesity. CD206 and CD11c show differential expression in CLS (white arrows) and FALC (orange arrow), but they show coexpress in cells between adipocytes (blue arrows). Blood vessels (blue) were stained with UEA-1 lectin. Scale bar: 100 μm. Row 1: epifluorescence microscopy. Rows 2–4: confocal microscopy.

An increase in proinflammatory cytokines is a hallmark of adipose tissue inflammation that is associated with metabolic dysfunction. Among a set of proinflammatory cytokines, *TNF*, *IL6*, *IL1B*, *IFNG*, and *CXCL8* (*IL8*) all were higher in DM compared with NDM VAT by bulk RNA-Seq. Among classically antiinflammatory cytokines, *IL10* was higher in DM and *IL5* was higher in NDM VAT (Figure 6A). In sorted ATM RNA-Seq data, all subtypes expressed *TNF*, *IL6*, *IL1B*, *IL8*, and *IL10* (Figure 6B and Supplemental Figure 3E). In CD206^+^ ATMs, *IL6* and *IL8* were increased compared with CD11c^+^ and DP ATMs. DP ATMs had increased *TNF* and *IL10* compared with both other ATM subtypes, and DP and CD11c^+^ ATMs had similarly high expression of *IL1B* compared with CD206^+^ ATMs. Analysis of cytokine interaction networks in sorted ATM RNA-Seq data support a prominent role for CSF1R (CD115) in CD206^+^ ATMs and IL1B in CD11c^+^ ATMs (Supplemental Figure 4).

We sorted ATMs based on CD206 expression to determine the in vitro cytokine secretion profile for CD206^+^ ATMs and CD206^–^ (CD11c^+^) ATMs. At baseline, cultured CD206^+^ and CD206^–^ ATMs secreted comparable levels of all cytokines except IL-5, which had very low levels in both subsets (Figure 6C). LPS-stimulated CD206^+^ ATM cultures, compared with non-LPS stimulated, contained significantly greater amounts of all cytokines except IFN-γ and IL-8, while in CD206^–^ ATMs all cytokines except IL-5 increased with LPS treatment (Figure 6D). IFN-γ was the only cytokine that was increased in CD206^–^ ATMs compared with CD206^+^ in the LPS condition. Together, these data suggest that CD206^+^ ATMs contribute to both proinflammatory and antiinflammatory cytokines in DM VAT.

### Human ATM subtypes localize to adipose tissue niches.

In murine obesity, recruited CD11c^+^ ATMs localize to crown-like structures (CLS). To investigate whether the ATM subtypes have unique localization to adipose tissue structures, we examined 2 fixed human VAT samples from patients with obesity who were immunostained for CD206 and CD11c. Confocal imaging revealed the presence of both CD206^+^ and CD11c^+^ ATMs in CLS, with bias toward a higher quantity of ATMs expressing only CD11c (Figure 6E). DP ATMs expressing CD206 and CD11c were identified throughout the tissue between adipocytes. Additionally, CD206^+^ and CD11c^+^ ATMs were found, with minimal coexpression, in vascularized fat-associated lymphoid clusters (FALCs), which are associated with tissue inflammation and antigen presentation (31). CD206^+^ ATMs were also present in greater quantity than CD11c^+^ ATMs in FALCs. Their unique localization suggests a role of both CD206^+^ and CD11c^+^ ATMs in adipose tissue niches relevant to maintenance of adipocyte function and potentiation of immune responses.

## Discussion

This study explores gene expression signatures in human ATM subtypes in obesity, and considering newly available transcriptomics datasets, comparative analyses may be useful tools for determining appropriate markers that identify ATM subsets. We compared these profiles with existing data sets and whole tissue gene expression in DM and NDM VAT and SAT in obesity, and we selectively evaluated surface proteins, intracellular lipids, and cytokine expression in ATM subtypes. Our data support an association between metabolic dysfunction and elevated VAT CD206^+^ and DP ATMs. While our study was not powered to detect correlations with sex, age, and BMI, we controlled for these potential confounders in post hoc statistical analyses. Larger studies will be required to determine the role of sex, age, BMI, and other clinical variables on ATM frequencies. We did not perform in vivo measures of systemic insulin resistance such as glucose tolerance testing in our human cohort, but we have demonstrated that our methods of categorizing DM using clinical diagnosis and HbA1c levels correlate strongly with molecular and cellular characteristics of adipose tissue (32–35), suggesting that DM is the main driver of the associations observed.

CD206^+^ ATMs were transcriptionally distinct from subtypes found by others to be associated with insulin resistance (9). Unique SR expression and lipid retention among ATM subtypes may reflect unique mechanisms of phagocytosis, lipid metabolism, and antigen presentation that directly influence tissue physiology. SR expression was prominent in CD206^+^ ATMs, but not DP or CD11c^+^ ATMs, and it was enriched in DM VAT. Notably, while lipid-laden DP and CD11c^+^ ATMs had lower expression of most SRs, *CLEC7A* and *OLR1* were increased in these subtypes and were also enriched in DM VAT. Lipid-laden ATMs expressed the lipid antigen presentation genes *CD1A-E* (Figure 4), consistent with ref. 9, which have been shown to interact with NKT cells to promote inflammation in diet-induced obesity in mice (36). Other features of CD11c^+^ ATMs, however, point to protective roles in adipose tissue. The lipid receptor TREM2, for example, is expressed in CD11c^+^ ATMs (Figure 4). *Trem2* deficiency in diet-induced obesity in mice was recently shown to eliminate CLS and infiltrating CD11c^+^ macrophages and to worsen insulin resistance (12, 37). Ours and other recent data support a model in which CD11c^+^ ATM subtypes localize to CLS, accumulating in response to changes in local lipid concentrations and obesity-related chemokines, becoming lipid-laden and protecting adipose tissue from lipotoxicity (2, 28, 38–40). Furthermore, significant ATM accumulation was observed in FALCs, particularly for CD206^+^ ATMs, leading us to speculate that FALCs provide a niche for proliferation of resident ATMs that modulate inflammation and activation of adaptive immune cells (41). The presence of CD11c^+^ ATMs also fits with a known association of FALCs with MHC II– and CD1-based antigen presentation and adipose tissue T cell activation by ATMs (26, 31).

Based on overall expression signatures and our finding that CD11c^+^ ATMs had the strongest overlap with the monocyte transcriptional signature in obesity, it is tempting to speculate that a lineage based on infiltrating monocytes in humans may first differentiate into CD11c^+^ ATMs that then later express CD206 and become DP ATMs. This would fit with a model in which, as for mice, humans have an infiltrating, monocyte-derived lineage of CD11c^+^ ATMs and a resident population of CD11c^−^ ATMs. Both monocyte-derived and resident ATMs may contribute to chronic inflammation through production of proinflammatory mediators, potentially due to dysfunction in their primary roles regulating lipid metabolism and tissue homeostasis.

Understanding the functional roles of all human ATM subtypes remains a central question in obesity. Our data show that gene expression signatures unique to each ATM subtype were enriched in DM adipose tissue. In mice, both M1-like CD11c^+^ and M2-like CD11c^−^ ATM subtypes increase in obesity (2, 42). Primarily M1-like ATMs have been associated with inflammation, adipose dysfunction, and insulin resistance in mice (3), but identification of M2 and mixed M1/M2 subtypes that are activated in obesity complicate this picture (43, 44). In humans, mixed M1/M2 types that are associated with insulin resistance also express proinflammatory mediators (7, 9, 10, 45). We found that CD206^+^ ATMs secreted both proinflammatory and antiinflammatory cytokines, with a profile that included IL-6, IL-8 and IL-10, whereas CD11c^+^ and DP ATMs secreted IL-1B and IL-10. MacSpectrum analysis of human ATM subtypes showed a higher proinflammatory index (MPI) for CD11c^+^ and DP ATMs, though subtypes were within 3 points of each other on the 100-point index. Divergent populations within the ATM subtypes studied here would account for mixed cytokine results, though further studies would be required to determine whether proinflammatory and antiinflammatory ATMs form distinct functional populations or whether ATMs primarily acquire a mixed M1/M2, obesity metabolically activated phenotype in human obesity and diabetes. Our results contribute to understanding of the significant heterogeneity in obesity-activated human ATMs and offer clues toward functional differences. These data support further investigation into the role of ATM subtypes in adipose tissue inflammation and dysfunction across the spectrum of phenotypic variation in human obesity.

## Methods

### Human participants.

Lean participants or obese participants undergoing bariatric surgery were recruited from the University of Michigan and the Ann Arbor Veterans Administration Hospital. Obesity was defined as BMI > 30 kg/m^2^ for at least 5 years. Obese participants were stratified into NDM and DM; DM was defined by clinical diagnosis requiring medication and hemoglobin A1c (HbA1c) ≥ 6.5%. NDM participants were defined by no clinical history of diabetes and HbA1c < 5.7% per American Diabetes Association criteria (Table 3). Metabolic syndrome criteria included diagnosis of DM, obstructive sleep apnea, hypertension, and dyslipidemia. Participants diagnosed with prediabetes or elevated HbA1c but not treated with diabetes-related medications, or those diagnosed with type 1 diabetes, were excluded. Mean HbA1c and fasting blood glucose were significantly elevated in obese DM participants compared with NDM. NDM participants had no clinical history of diabetes and normal percentage of HbA1c. Additionally, compared with lean and obese NDM, obese DM participants had an increased frequency of dyslipidemia and use of statin, metformin, insulin, and sulfonylurea medications. Adipose tissues (VAT from the greater omentum and SAT from abdominal skin incisions) were collected at the beginning of surgery and processed immediately.

### Adipose tissue processing and SVC isolation.

For flow cytometry analyses, single-cell suspensions of SVCs were obtained from adipose tissue after fine mincing by hand using surgical scissors (DR Instruments, 4SB), digesting in 1 mg/mL collagenase II (Invitrogen, 17101015) for 1 hour with agitation on an orbital shaker at 37°C, 100 μm straining, NH_4_Cl RBC lysis, and quantification by hemocytometer. For sorting, we optimized SVC isolations to maximize yield while decreasing processing time, using the above method with 3 mg/mL collagenase II and digesting for 30 minutes in 10 mL within a 50 mL conical tube at 0.2 grams/mL. Digests were agitated on a rocker, shaking tubes by hand at 5-minute intervals. Yields by this method were 0.5 × 10^6^ to 1 × 10^6^ SVCs per gram. Digest and wash buffer was HBSS with calcium/magnesium (Thermo Fisher Scientific) and 0.5% BSA (Sigma-Aldrich).

### Antibodies and immunostaining.

Antibodies and stains for flow cytometry analysis and sorting were: CD45 PerCP-Cy5.5 (Thermo Fisher Scientific, 45-0459-41, RRID:AB_10718244, clone Hl30), CD64 PE (BioLegend, 305008, RRID:AB_314492, clone 10.1), CD11c PE-Cy7 (BioLegend, 301608, RRID:AB_389351, clone 3.9), CD206 APC-Cy7 (BioLegend, 321119, RRID:AB_2144932, clone 15-2), LipidTOX Deep Red Neutral Lipid Stain (Invitrogen, H34477), LIVE/DEAD Fixable Violet Dead Cell Stain (Invitrogen, L34955), and HLA-DR PC5 (Beckman Coulter, IM2659U, Immu-357). For CD1 flow staining, VAT-derived SVCs from 2 independent participants with obesity and NDM, 1 male and 1 female, were stained with CD1B APC (BioLegend, 329109, RRID: AB_2563952), CD1C APC (BioLegend, 331523, RRID: AB_10718511), or CD1D APC (BioLegend, 350307 RRID AB_10642029), using Human TruStain FcX (BioLegend, 422301) for Fc receptor blocking with Human TruStain FcX (BioLegend, 422301) was performed. Cells in suspension were stained for 20–30 minutes at 4°C.

For tissue imaging, human VAT from 2 independent participants with obesity and DM was cut into approximately 1 × 1 × 0.4 cm pieces and fixed in PBS/1% PFA for 1 hour at room temperature (RT) with gentle rocking. Samples were blocked with PBS/5% goat serum/1% BSA (blocking buffer) for 1 hour at RT, incubated overnight at 4°C with primary antibodies diluted in blocking buffer, washed 3 × 15 minutes with PBS, incubated for 1 hour at RT protected from light with secondary antibodies and blood vessel stain diluted in PBS/1% BSA, and washed 3 × 15 minutes in PBS. Primary antibodies included CD206 (BioLegend, 321101, RRID:AB_571922, clone 15-2) and CD11c (Abcam, ab52632, RRID:AB_2129792, clone EP1347Y). Secondary antibodies included goat anti–mouse IgG (H+L) Highly Cross-Adsorbed, AlexaFluor 488 (Invitrogen, A-11029, RRID: AB_2534088), and goat anti–rabbit IgG (H+L) Highly Cross-Adsorbed, AlexaFluor 568 (Invitrogen, A-11036, RRID:AB_10563566). Ulex Europaeus Agglutinin I–DyLight 649, (Vector Laboratories, DL-1068) was used to stain blood vessels. Goat serum was obtained from Jackson ImmunoResearch (005-000-121, RRID:AB_2336990).

### Microscopy.

Epifluorescence microscopy was performed using an Olympus iX81 microscope. The objective used was the Olympus UPlan SApo 20× (N.A. 0.75). Confocal microscopy was performed using a Nikon A1 confocal microscope in the Imaging Lab at the Michigan Diabetes Research Center (MDRC) Microscopy, Imaging, and Cellular Physiology Core (MICPC) (RRID:SCR_015118). The objectives used were the Nikon Plan Apo 20X DIC M N2 (N.A. 0.75) and the Nikon Plan Apo VC 60XA WI DIC N2 (N.A. 1.20). Adipose tissue was placed onto Nunc Lab-Tek II Chambered Coverglass No. 1.5 (Thermo Fisher Scientific, 155360) for microscopy and image acquisition. ImageJ software (NIH) was used for contrast, brightness, and pseudocolor adjustments.

### Flow cytometry and sorting.

All flow cytometers and sorters used in these studies were located in and maintained by the University of Michigan Biomedical Research Flow Cytometry Core. Data from stained cells were collected for flow cytometry analysis using BD FACSCanto II and LSRFortessa flow cytometers. Flow cytometry data were analyzed using FlowJo software v10.1 (Tree Star Inc.), using manual compensation and fluorescence minus one (FMO) controls to guide gating. Initial gates identified live, nonaggregated, CD45^+^ leukocytes (Figure 1A). ATMs for RNA-Seq were sorted on a Beckman Coulter MoFlo Astrios into 50% FBS in phosphate buffered saline. For in vitro cytokine assays, obese DM SVCs were isolated, stained, and immediately sorted on a BD FACSAria III sorter, collecting 2 populations of ATMs: CD45^+^CD64^+^CD206^+^ (CD206^+^) and CD45^+^CD64^+^CD206^−^ (CD206^−^).

### Cell processing for RNA-Seq.

Sorted cells were centrifuged at 400*g* for 10 minutes at 4°C and supernatant removed. Up to 250,000 cells were lysed by vortexing pellets in 1 mL 1% β-mercaptoethanol/RLT buffer (Qiagen), and lysate was stored at –80°C for later processing. Samples were placed at 37°C just until thawed; they were then run through QIAshredder columns for sample homogenization. RNA from the QIAshredder flow-through was purified with the RNeasy Mini Kit (Qiagen). The University of Michigan Sequencing Core confirmed RNA integrity on an Agilent 2100 Bioanalyzer, and samples were then processed with the Pico Input Mammalian SMARTer Stranded Total RNA-Seq Kit (Clontech).

### Cytokine assays.

Sorted CD206^+^ and CD206^−^ ATMs were each plated at 5000 cells/well on a U-bottom 96-well culture plate (Falcon, 353077), and incubated at 37°C with 5% CO_2_ for 21 hours in Thermo FIsher Scientific DMEM/F12 (Ham’s) (1:1), containing 15 mm HEPES (+L-Glutamine) (Thermo Fisher Scientific, 11330-032), with 10% HI-FBS (Thermo Fisher Scientific, 10082) and 1% penicillin/streptomycin (Thermo Fisher Scientific, 15140-122). Reagents for stimulation treatment were diluted in fresh culture medium, and cells were either untreated or treated with LPS (Sigma Aldrich, L2630; lipopolysaccharides from *E. coli* O111:B4) at 10 ng/μL or 100ng/μL. After 20 hours of stimulation, medium was removed and frozen at –70°C. Medium was submitted to the MDRC Chemistry Laboratory for the High Sensitivity T-Cell Multiplex Assay (Millipore Sigma, HSTCMAG-28SK).

### RNA-Seq and data pipeline.

RNA-Seq reads files obtained from the Illumina Hi-Seq platform at the University of Michigan Advanced Genomics Core were downloaded by the UM Bioinformatics Core, which processed the data as follows. Reads files were concatenated into a single .fastq file for each sample. Quality of raw reads data for each sample was checked using FastQC (v0.11.3). Low-quality bases were trimmed from individual reads using CutAdapt. The Tuxedo Suite software package was used for alignment, differential expression analysis, and postanalysis diagnostics (46–48). Briefly, reads were aligned to the reference mRNA and lncRNA transcriptome (UCSC hg19) using TopHat (v2.0.13) and Bowtie (v2.2.1.). Default parameter settings were used for alignment, with the exception of: “—b2-very-sensitive” telling the software to spend extra time searching for valid alignments. FastQC was used a second time for quality control (after alignment), to ensure that only high-quality data would be input to expression quantitation and differential expression analysis. Cufflinks/CuffDiff (v2.2.1) was used for expression quantitation, normalization, and differential expression analysis, using UCSC hg19.fa as the reference genome sequence. The following parameter settings were used: “—multi-read-correct” to adjust expression calculations for reads that map in more than 1 locus, and “—compatible-hits-norm” and “—upper-quartile –norm” for normalization of expression values. Diagnostic plots were generated using the CummeRbund package. Locally developed scripts were used to format and annotate the differential expression data output from CuffDiff. Briefly, DE genes and transcripts were identified based on 3 criteria: test status was set as “OK”, FDR ≤ 0.05, and fold change ≥ ± 1.5. DAVID (v6.7) was used for enrichment analysis of the set of DE genes to identify significantly enriched functional categories.

### Gene set and MacSpectrum analyses.

Gene set enrichment analysis (Molecular Signatures Database) (RRID:SCR_003199) was used to identify signatures in our data that overlapped with existing datasets. iPathways software (Advaita) was used for pathway analysis with FDR ≤ 0.05. Control sets of 20 genes for ATMs and adipose tissues were generated by randomly sorting all genes in each data set for which at least 1 sample had expression > 10 FPKM. Ten iterations of random sets were used to establish frequencies of genes increased in the sample of interest expected by chance. For analysis of antigen presentation, HLA-D genes shown had significant differential expression for at least 1 comparison (Figure 4). For MacSpectrum analysis, gene expression data were filtered for DE genes in the comparison of CD206^+^ with CD11c^+^ ATMs and for expression of at least 10 FPKM. For genes fitting these criteria, FPKM values for all 3 ATM subtypes were run in MacSpectrum v1.0.1 at https://macspectrum.uconn.edu (run January 2021) with the “Human” setting to obtain MPI and AMPI values

### Data and resources.

The data discussed in this publication have been deposited in NCBI’s Gene Expression Omnibus (49) and are accessible through GEO Series accession no. GSE189849 (https://www.ncbi.nlm.nih.gov/geo/query/acc.cgi?acc=GSE189849).

### Statistics.

Statistical tests included Spearman correlation, 1-way ANOVA with post hoc test for linear trends or Holm-Sidak multiple comparisons post hoc test, unpaired 2-tailed *t* test with Welch’s correction, and paired 2-tailed *t* test (Wilcoxon matched-pairs signed-rank test) for comparisons between adipose depots. Fisher’s exact test was used to compare dichotomous patient demographic variables. Least squares multiple regression was used with HbA1c (dependent), age, sex, BMI, and frequency of cell type. A *P* value less than 0.05 was considered significant. Statistical tests were performed using GraphPad Prism (v8.4.3).

### Study approval.

Human participants participated in this study with IRB approval at the University of Michigan (HUM00074075), and written informed consent was obtained prior to inclusion in this study.

## Author contributions

LAM and CNL conceived and designed experiments and wrote and edited the paper with input from all authors. LAM, KWC, LMG, NAB, CGF, and APE processed tissue and performed experiments. LAM, NK, SL, SR, IR, RWO, and CNL analyzed and interpreted data.

## Supplementary Material

Supplemental data

## Figures and Tables

**Table 1:**
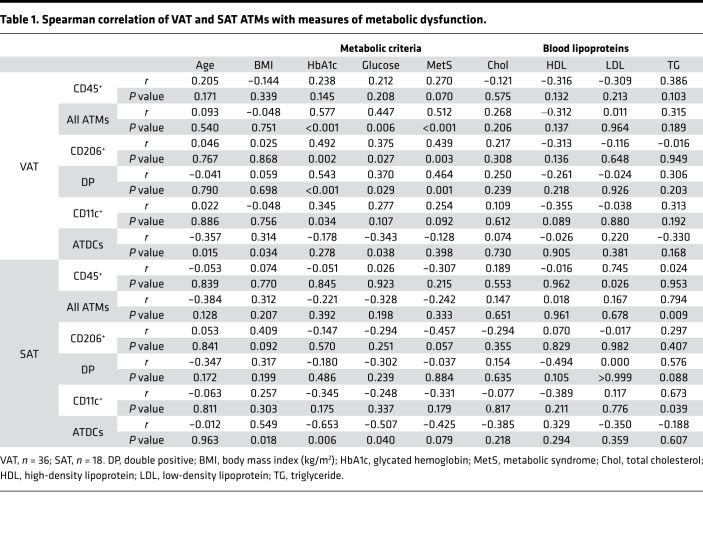
Spearman correlation of VAT and SAT ATMs with measures of metabolic dysfunction.

**Table 2:**
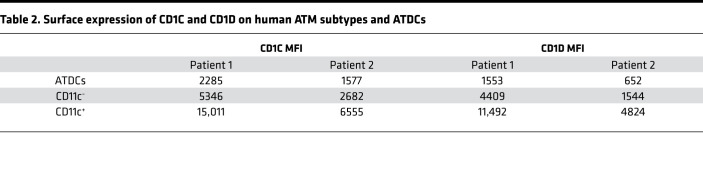
Surface expression of CD1C and CD1D on human ATM subtypes and ATDCs

**Table 3:**
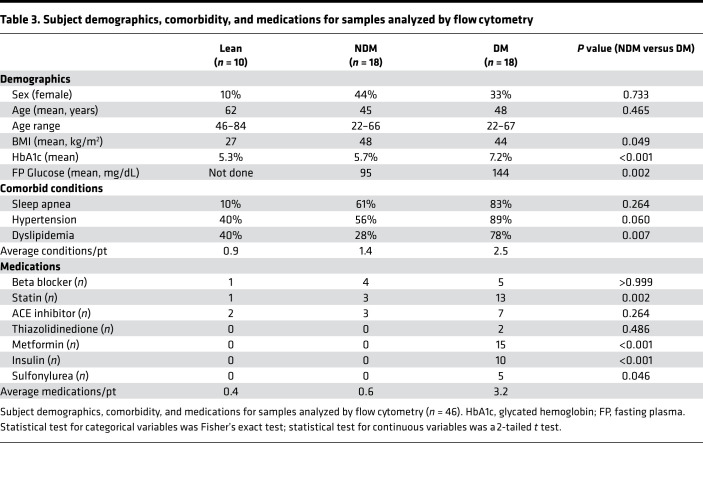
Subject demographics, comorbidity, and medications for samples analyzed by flow cytometry

## Version 1 01/06/2022

In-Press Preview

## Version 2 02/08/2022

Electronic publication

## References

[1] Kruglikov IL (2020). Obesity and diabetes as comorbidities for COVID-19: underlying mechanisms and the role of viral-bacterial interactions. Elife.

[2] Muir LA (2018). Frontline science: rapid adipose tissue expansion triggers unique proliferation and lipid accumulation profiles in adipose tissue macrophages. J Leukoc Biol.

[3] Lumeng CN (2007). Increased inflammatory properties of adipose tissue macrophages recruited during diet-induced obesity. Diabetes.

[4] Lumeng CN (2007). Obesity induces a phenotypic switch in adipose tissue macrophage polarization. J Clin Invest.

[5] Cho KW (2016). Adipose tissue dendritic cells are independent contributors to obesity-induced inflammation and insulin resistance. J Immunol.

[6] Weisberg SP (2003). Obesity is associated with macrophage accumulation in adipose tissue. J Clin Invest.

[7] Bourlier V (2008). Remodeling phenotype of human subcutaneous adipose tissue macrophages. Circulation.

[8] Aron-Wisnewsky J (2009). Human adipose tissue macrophages: m1 and m2 cell surface markers in subcutaneous and omental depots and after weight loss. J Clin Endocrinol Metab.

[9] Wentworth JM (2010). Pro-inflammatory CD11c+CD206+ adipose tissue macrophages are associated with insulin resistance in human obesity. Diabetes.

[10] Fjeldborg K (2014). Human adipose tissue macrophages are enhanced but changed to an anti-inflammatory profile in obesity. J Immunol Res.

[11] Gautier EL (2012). Gene-expression profiles and transcriptional regulatory pathways that underlie the identity and diversity of mouse tissue macrophages. Nat Immunol.

[12] Jaitin DA (2019). Lipid-associated macrophages control metabolic homeostasis in a Trem2-dependent manner. Cell.

[13] Vijay J (2020). Single-cell analysis of human adipose tissue identifies depot and disease specific cell types. Nat Metab.

[14] Russo L, Lumeng CN (2018). Properties and functions of adipose tissue macrophages in obesity. Immunology.

[15] Singer K (2015). Differences in Hematopoietic Stem Cells Contribute to Sexually Dimorphic Inflammatory Responses to High Fat Diet-induced Obesity. Metabolism.

[16] Varghese M (2019). Sex Differences in Inflammatory Responses to Adipose Tissue Lipolysis in Diet-Induced Obesity. Endocrinology.

[17] Rasouli N (2009). Association of scavenger receptors in adipose tissue with insulin resistance in nondiabetic humans. Arterioscler Thromb Vasc Biol.

[18] Cai L (2012). Scavenger receptor CD36 expression contributes to adipose tissue inflammation and cell death in diet-induced obesity. PLoS One.

[19] Nicholls HT (2011). Hematopoietic cell-restricted deletion of CD36 reduces high-fat diet-induced macrophage infiltration and improves insulin signaling in adipose tissue. Diabetes.

[20] Kennedy DJ (2011). A CD36-dependent pathway enhances macrophage and adipose tissue inflammation and impairs insulin signalling. Cardiovasc Res.

[21] Yona S (2013). Fate mapping reveals origins and dynamics of monocytes and tissue macrophages under homeostasis. Immunity.

[22] Hassnain Waqas SF (2017). Adipose tissue macrophages develop from bone marrow-independent progenitors in Xenopus laevis and mouse. J Leukoc Biol.

[23] Hulsmans M (2012). Interleukin-1 receptor-associated kinase-3 is a key inhibitor of inflammation in obesity and metabolic syndrome. PLoS One.

[24] Li C (2019). Single cell transcriptomics based-MacSpectrum reveals novel macrophage activation signatures in diseases. JCI Insight.

[25] Morris DL (2013). Adipose tissue macrophages function as antigen-presenting cells and regulate adipose tissue CD4+ T cells in mice. Diabetes.

[26] Cho KW (2014). An MHC II-dependent activation loop between adipose tissue macrophages and CD4+ T cells controls obesity-induced inflammation. Cell Rep.

[27] Shapiro H (2013). Adipose tissue foam cells are present in human obesity. J Clin Endocrinol Metab.

[28] Hill DA (2018). Distinct macrophage populations direct inflammatory versus physiological changes in adipose tissue. Proc Natl Acad Sci U S A.

[29] Russo L (2020). Cholesterol 25-hydroxylase (CH25H) as a promoter of adipose tissue inflammation in obesity and diabetes. Mol Metab.

[30] Eisenberg E, Levanon EY (2013). Human housekeeping genes, revisited. Trends Genet.

[31] Bénézech C (2015). Inflammation-induced formation of fat-associated lymphoid clusters. Nat Immunol.

[32] Muir LA (2016). Adipose tissue fibrosis, hypertrophy, and hyperplasia: correlations with diabetes in human obesity. Obesity (Silver Spring).

[33] Baker NA (2017). Diabetes-specific regulation of adipocyte metabolism by the adipose tissue extracellular matrix. J Clin Endocrinol Metab.

[34] Cotillard A (2014). Adipocyte size threshold matters: link with risk of type 2 diabetes and improved insulin resistance after gastric bypass. J Clin Endocrinol Metab.

[35] O’Connell J (2010). The relationship of omental and subcutaneous adipocyte size to metabolic disease in severe obesity. PLoS One.

[36] Satoh M (2016). Adipocyte-specific CD1d-deficiency mitigates diet-induced obesity and insulin resistance in mice. Sci Rep.

[37] Liu C (2019). TREM2 regulates obesity-induced insulin resistance via adipose tissue remodeling in mice of high-fat feeding. J Transl Med.

[38] Xu X (2013). Obesity activates a program of lysosomal-dependent lipid metabolism in adipose tissue macrophages independently of classic activation. Cell Metab.

[39] Kosteli A (2010). Weight loss and lipolysis promote a dynamic immune response in murine adipose tissue. J Clin Invest.

[40] Prieur X (2011). Differential lipid partitioning between adipocytes and tissue macrophages modulates macrophage lipotoxicity and M2/M1 polarization in obese mice. Diabetes.

[41] Chakarov S (2019). Two distinct interstitial macrophage populations coexist across tissues in specific subtissular niches. Science.

[42] Nguyen MTA (2007). A subpopulation of macrophages infiltrates hypertrophic adipose tissue and is activated by free fatty acids via Toll-like receptors 2 and 4 and JNK-dependent pathways. J Biol Chem.

[43] Kratz M (2014). Metabolic dysfunction drives a mechanistically distinct proinflammatory phenotype in adipose tissue macrophages. Cell Metab.

[44] Lindhorst A (2021). Adipocyte death triggers a pro-inflammatory response and induces metabolic activation of resident macrophages. Cell Death Dis.

[45] Zeyda M (2010). Newly identified adipose tissue macrophage populations in obesity with distinct chemokine and chemokine receptor expression. Int J Obes (Lond).

[46] Langmead B (2009). Ultrafast and memory-efficient alignment of short DNA sequences to the human genome. Genome Biol.

[47] Trapnell C (2009). TopHat: discovering splice junctions with RNA-seq. Bioinformatics.

[48] Trapnell C (2013). Differential analysis of gene regulation at transcript resolution with RNA-seq. Nat Biotechnol.

[49] Edgar R (2002). Gene expression omnibus: NCBI gene expression and hybridization array data repository. Nucleic Acids Res.

