# Supplementary material for: Human CD206+ macrophages associate with diabetes and adipose tissue lymphoid clusters
Source: JCI Insight. 2022 Feb 8;7(3):e146563. doi: 10.1172/jci.insight.146563 (PMC8855803; doi:10.1172/jci.insight.146563)
Supplement: Supplemental data [file jciinsight-7-146563-s111.pdf]

Supplemental Figure 1

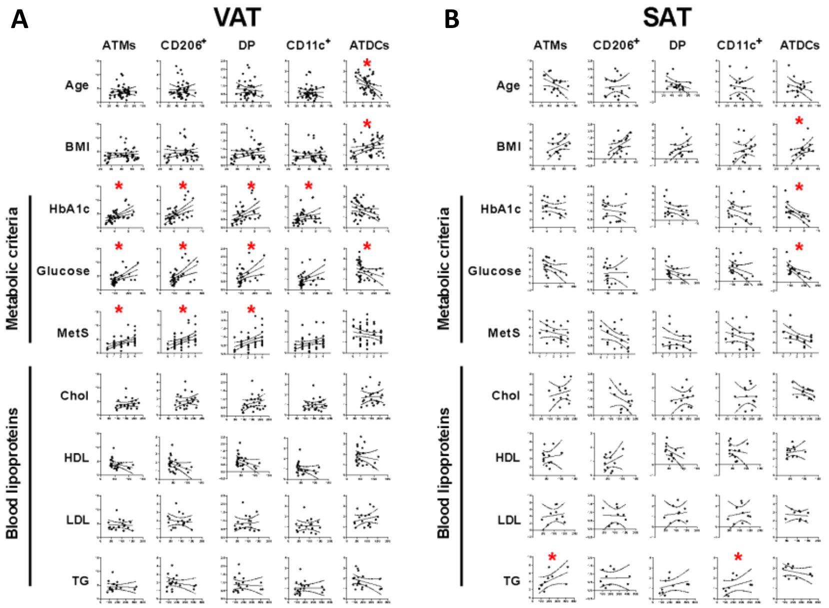

C

DV: HbA1c	IV	β	SE	t-Statistic	P value	R^2
Total ATMs	Cell Frequency	0.293	0.082	3.589	**0.001**	0.295
	Age	0.006	0.013	0.479	0.635	
	Female	0.196	0.358	0.547	0.588	
	BMI	0.007	0.019	0.384	0.704	
CD206$^+$ ATMs	Cell Frequency	0.545	0.151	3.604	**0.001**	0.297
	Age	0.008	0.014	0.598	0.554	
	Female	0.157	0.360	0.435	0.667	
	BMI	0.012	0.020	0.622	0.539	
CD11c$^+$ ATMs	Cell Frequency	0.397	0.289	1.372	0.179	0.073
	Age	0.008	0.016	0.526	0.602	
	Female	-0.048	0.418	0.115	0.909	
	BMI	0.009	0.023	0.416	0.680	
DP ATMs	Cell Frequency	0.941	0.358	2.630	**0.013**	0.190
	Age	0.010	0.015	0.687	0.497	
	Female	0.069	0.386	0.179	0.859	
	BMI	0.005	0.021	0.249	0.805	
ATDCs	Cell Frequency	-0.400	0.244	1.642	0.110	0.099
	Age	0.000	0.016	0.023	0.982	
	Female	-0.242	0.379	0.638	0.528	
	BMI	0.013	0.022	0.585	0.562	

DV, dependent variable; HbA1c, glycated hemoglobin; IV, independent variable; β, coefficient; SE, standard error

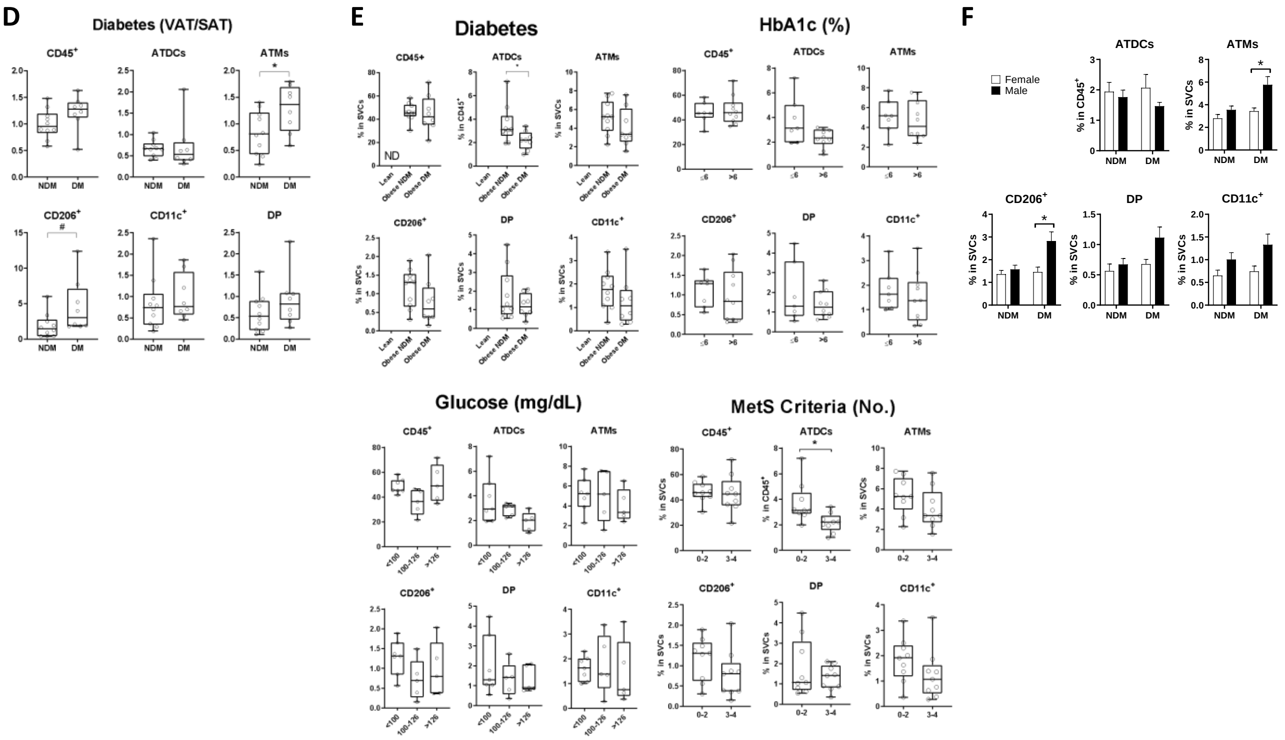

Supplemental Figure 1. Correlation and categorical analyses of ATMs and ATDCs. (**A**)-(**B**) Correlation scatter plots for ATM and ATDC frequencies and clinical measures for VAT and SAT. Best fit lines are shown based on linear regression, and dotted lines indicate the 95% confidence bands. Red asterisks indicate p<0.05 by Spearman correlation. (**C**) Multiple regression results examining VAT ATMs and ATDCs as predictors of HbA1c adjusting for age, sex, and BMI (**D**) Subjects stratified by diagnosis of diabetes, with indicated SVC frequencies in VAT normalized to frequencies in SAT for each individual. (**E**) SAT SVC frequencies in patients stratified by clinical criteria. (**F**) Subjects stratified by diagnosis of diabetes and sex. NDM female n=9; NDM male n=17; DM female n=6; DM male n=12.

Supplemental Figure 2

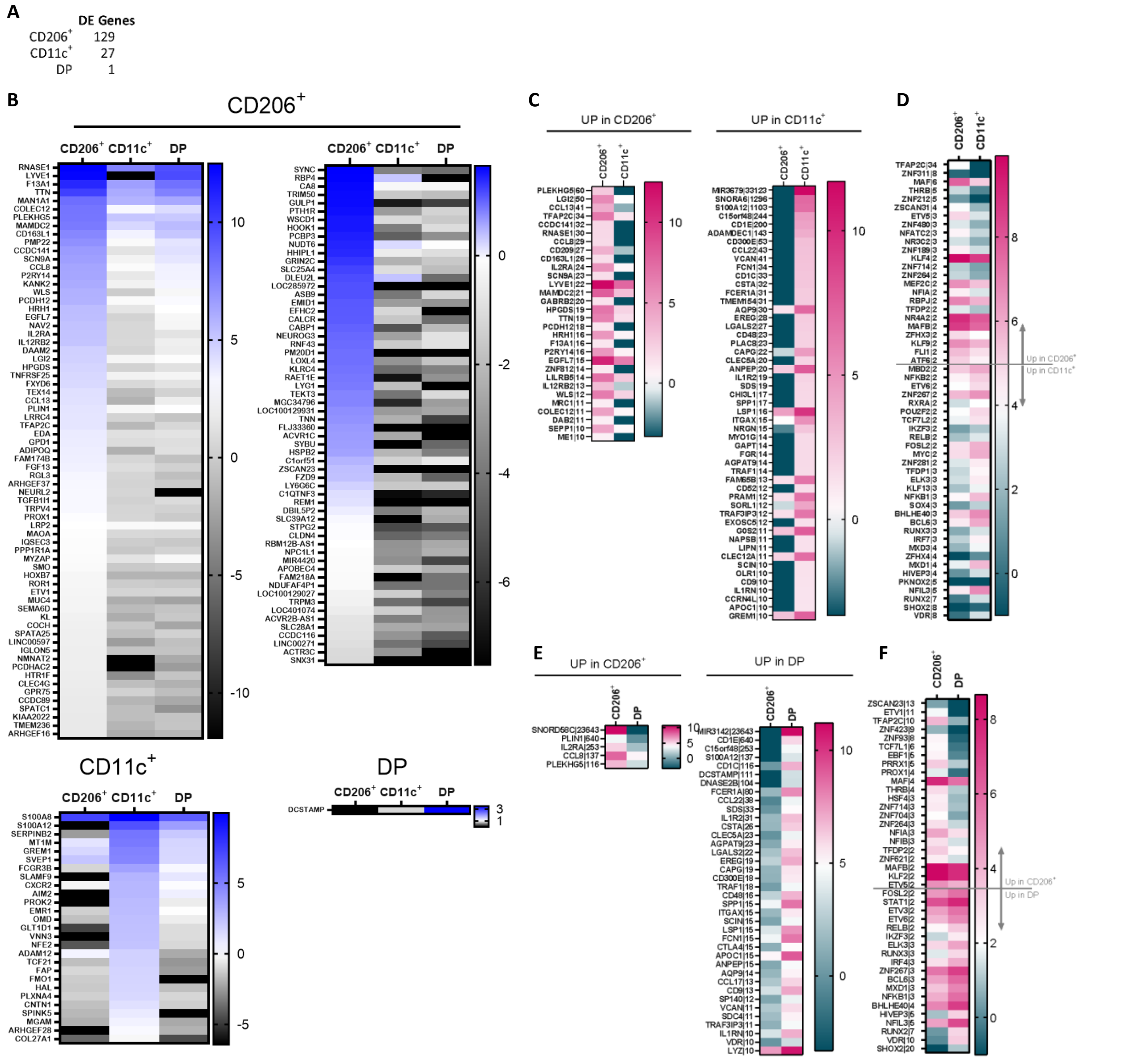

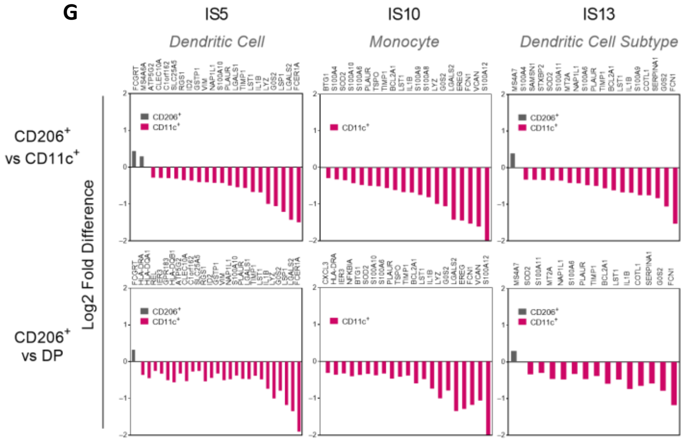

Supplemental Figure 2. Top differentially expressed genes in human ATM subtypes and overlap with published data. (**A**) Number of unique DE genes increased for each ATM subtype compared to both other subtypes, with at least five-fold difference in expression. (**B**) DE genes up in each subtype compared to both other subtypes, with at least five-fold difference in expression. (**C**) The most highly expressed DE genes with at least 10-fold change comparing CD206^{+} to CD11c^{+} ATMs. (**D**) DE transcription factors with the highest fold change comparing CD206^{+} ATMs to CD11c^{+} ATMs. (**E**) The most highly expressed DE genes with at least 10-fold change comparing CD206^{+} to DP ATMs. (**F**) DE transcription factors with the highest fold change comparing CD206^{+} ATMs to DP ATMs. (**G**) Overlap of human ATM subtypes with putative dendritic cells and monocyte gene expression from Vijay et al. 2020. Data shown as log2 (FPKM) unless otherwise indicated.

Supplemental Figure 3

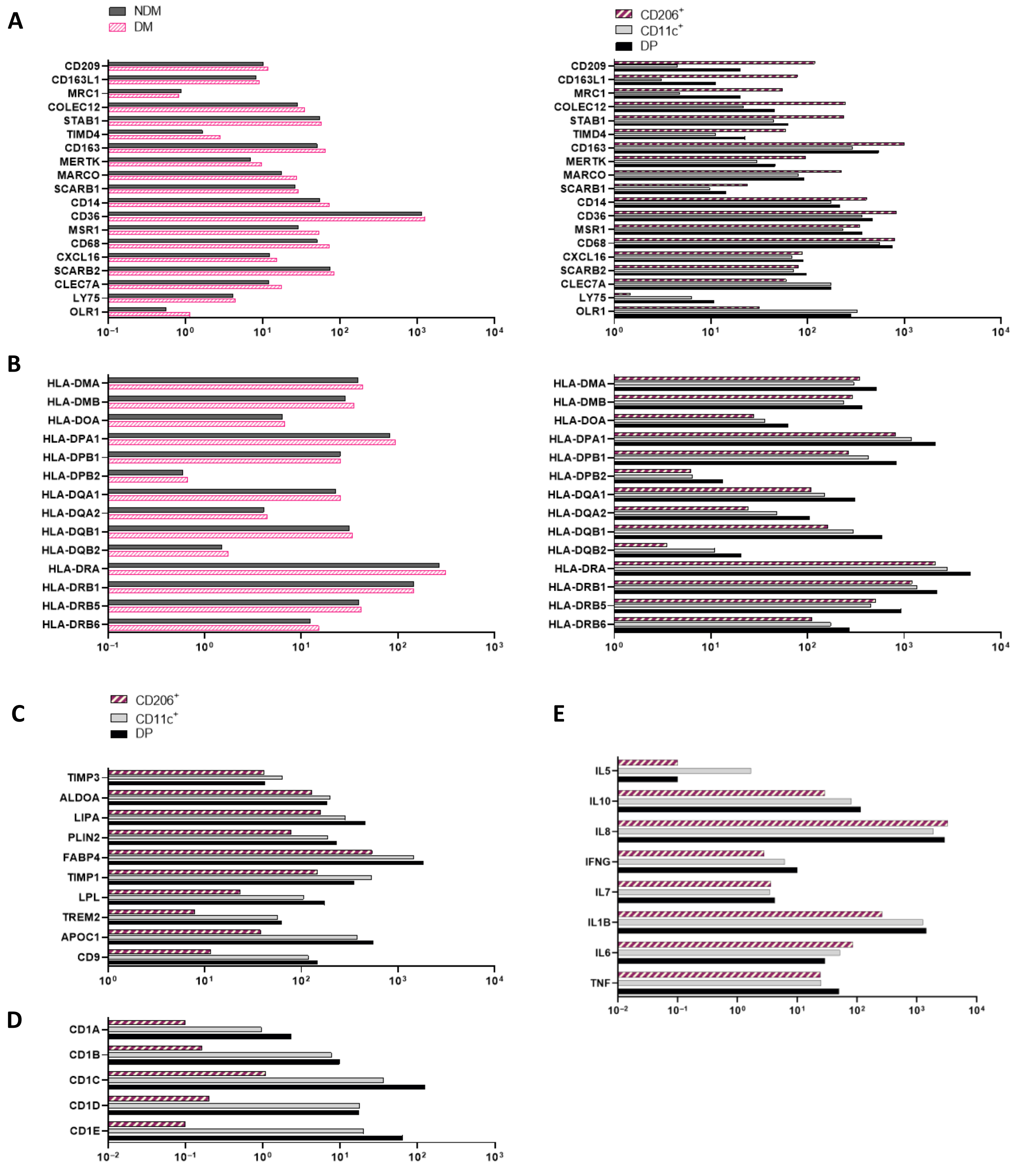

Supplemental Figure 3. Gene set expression shown in mean FPKM for VAT and ATM subtypes. Gene sets represent scavenger receptors (**A**), HLA-D genes (**B**), lipid-laden macrophages (**C**), lipid antigen presentation (**D**), and cytokines (**E**).

Supplemental Figure 4

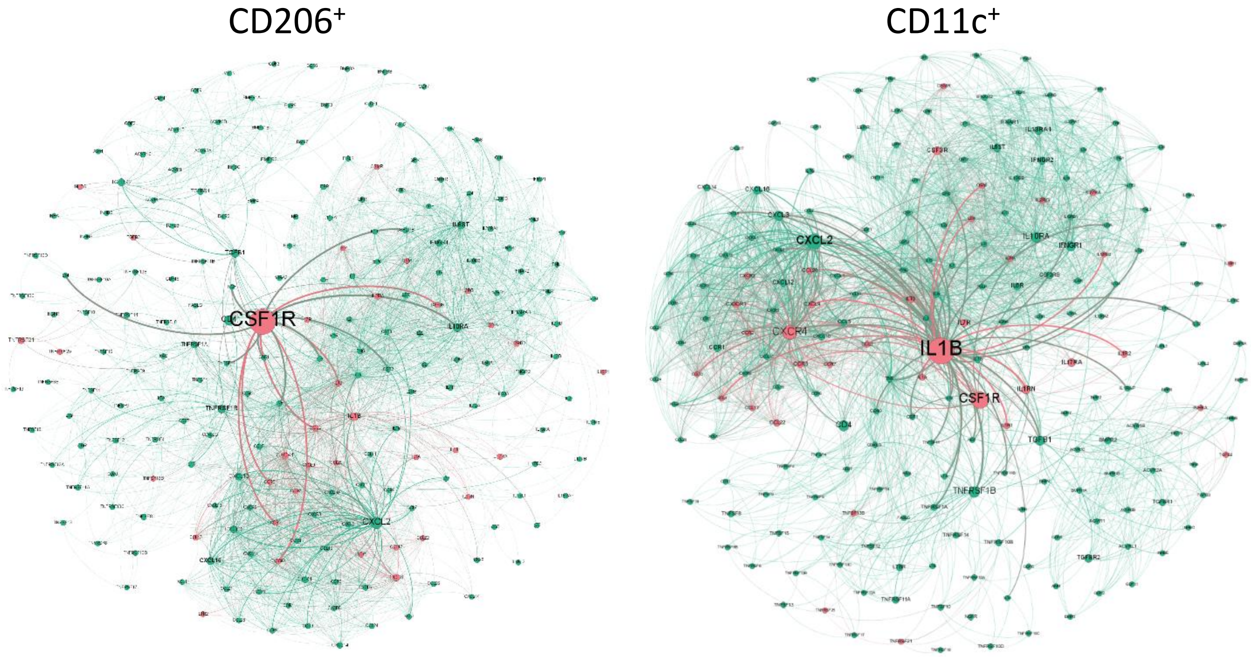

Cytokine interaction networks in two prominent human visceral ATM subtypes in obesity

Supplemental Figure 4. Supplemental analysis related to Figure 6. Cytokine gene expression networks constructed for CD206+ vs. CD11c+ ATMs. Nodes are genes, where the size of the node indicates relative expression. Edges between genes represent a known protein-protein interaction, where edge weight is the average of each pair of genes' expressions. Red nodes indicate DE genes in the networks.
